# Identification of Human MicroRNA-Like Sequences Embedded within the Protein-Encoding Genes of the Human Immunodeficiency Virus

**DOI:** 10.1371/journal.pone.0058586

**Published:** 2013-03-08

**Authors:** Bryan Holland, Jonathan Wong, Meng Li, Suraiya Rasheed

**Affiliations:** Laboratory of Viral Oncology and Proteomics Research, Keck School of Medicine, University of Southern California, Los Angeles, California, United States of America; George Mason University, United States of America

## Abstract

**Background:**

MicroRNAs (miRNAs) are highly conserved, short (18–22 nts), non-coding RNA molecules that regulate gene expression by binding to the 3′ untranslated regions (3′UTRs) of mRNAs. While numerous cellular microRNAs have been associated with the progression of various diseases including cancer, miRNAs associated with retroviruses have not been well characterized. Herein we report identification of microRNA-like sequences in *coding* regions of several HIV-1 genomes.

**Results:**

Based on our earlier proteomics and bioinformatics studies, we have identified 8 cellular miRNAs that are predicted to bind to the mRNAs of multiple proteins that are dysregulated during HIV-infection of CD4+ T-cells *in vitro*. *In silico* analysis of the full length and mature sequences of these 8 miRNAs and comparisons with all the genomic and subgenomic sequences of HIV-1 strains in global databases revealed that the first 18/18 sequences of the *mature* hsa-miR-195 sequence (including the short *seed* sequence), matched perfectly (100%), or with one nucleotide mismatch, *within* the envelope (*env*) genes of five HIV-1 genomes from Africa. In addition, we have identified 4 other miRNA-like sequences (hsa-miR-30d, hsa-miR-30e, hsa-miR-374a and hsa-miR-424) within the *env* and the *gag-pol* encoding regions of several HIV-1 strains, albeit with reduced homology. Mapping of the miRNA-homologues of *env* within HIV-1 genomes localized these sequence to the functionally significant variable regions of the *env* glycoprotein gp120 designated V1, V2, V4 and V5.

**Conclusions:**

We conclude that microRNA-like sequences are embedded within the protein-encoding regions of several HIV-1 genomes. Given that the V1 to V5 regions of HIV-1 envelopes contain specific, well-characterized domains that are critical for immune responses, virus neutralization and disease progression, we propose that the newly discovered miRNA-like sequences within the HIV-1 genomes may have evolved to self-regulate survival of the virus in the host by evading innate immune responses and therefore influencing persistence, replication and/or pathogenicity.

## Introduction

MicroRNAs (miRNAs) are highly conserved, naturally occurring, 18–22 nucleotides long, noncoding RNA molecules that are processed from long precursor transcripts (pre-miRNAs). The cellular miRNAs are transcribed by the same transcription factors and RNA polymerase II that control the transcription of protein-encoding messenger RNAs (mRNAs) [1]. The pre-miRNA transcripts are processed into mature miRNAs by complex molecular processes involving cleavage by RNaseIII-like enzyme Drosha in the nucleus, followed by their transport to the cytoplasm mediated by Exportin-5 and a second cleavage by Dicer, an RNAse III-like enzyme. One strand of this double-stranded miRNA forms the “mature” 18–22 nucleotide sequence, which becomes a part of a multi-protein RNA-induced silencing-complex (RISC) that guides it to the mRNA target site. The second strand is believed to be degraded [1].

The human genome contains >1,500 miRNA genes that have been predicted or experimentally shown to play critical roles in normal cellular functions, such as maintaining homeostasis, and regulating or modulating viral and cellular gene expression. Specific alterations in gene or protein expression profiles change the direction of normal cells toward the development of cancer and other disorders [2], [3]. An important post-transcriptional regulatory step in gene expression is that the 5′ ends of miRNAs can base-pair with the complementary sequences in the 3′ untranslated regions (UTRs) of their target mRNAs and suppress translational capacities of those mRNAs [4]. While the exact mechanism of miRNA-mediated regulation of the mRNA targets is still not fully understood, two aspects of the gene-expression controls are noteworthy. First, a short nucleotide sequence (nucleotides 2–7 from the 5′ end of a miRNA), referred to as the ‘*seed sequence’,* base-pairs completely and continuously with its target mRNA; next, this initial binding of the miRNA with its *seed sequence* allows complete or incomplete binding of the rest of the mature miRNA sequence with the target mRNA. Thus, gene repression by miRNA appears to depend primarily on complete base-pairing of their *seed sequences* with its complementary mRNA target; however, sometimes an incomplete base-pairing of the rest of the miRNA sequence also destabilizes the target transcript, represses the translation of the protein or influences degradation of the target mRNA [5]. Also, one miRNA can interact with multiple mRNA targets and a single mRNA can be regulated by multiple miRNAs. Upregulation or downregulation of a given miRNA can dysregulate protein expression profiles and therefore result in disruption of normal biological processes such as cell proliferation, development, differentiation, apoptosis, and signal transduction [4], [6], [7].

The bulk of well-characterized miRNAs are either from cellular genomes or from several DNA *viral genomes* and both can impact expression of cellular and viral genes respectively in infected cells [8]. Recently, a retrovirus, the bovine leukemia virus (BLV), has been shown to encode a conserved cluster of miRNAs that are transcribed by RNA polymerase III which mimics miRNAs involved in B-cell cancers [9].

*In silico* studies have predicted that the genomes of the human immunodeficiency virus-1 (HIV-1) may contain *target sites* for binding of *cellular miRNAs* [10]. For instance, hsa-miR-29a is reported to *target the nef region* of the HIV-1 genome [11]. Researchers have also shown that cellular miRNA expression patterns in HIV-1 infected peripheral blood mononuclear cells are altered post-infection [12], [13]. A recent report proposed that altered cellular miRNA profiles of HIV-infected cells could be used as early indicators of host cellular dysfunctions [14]. The existence of HIV-1 vmiRNAs, that may regulate both viral and host gene expression has also been suggested [15].

Based on the RNA structure and folding parameters consistent with cellular miRNA molecules, *five HIV-1-encoded miRNAs have been predicted* computationally [16]. However, there has been disagreement about the existence of vmiRNAs in HIV-1 genomes [17]. Currently, three HIV-1-encoded vmiRNAs have been listed in miRBase: miR-H1, miR-N367 and miR-TAR-5p/3p. These vmiRNAs have been reported to *target “viral” transcripts,* as in the case of miR-N367 [18], and host cellular factors for miR-H1 and miR-TAR-5p/3p [19], [20]. More recent research seems to support that miRNAs are in fact present in the viral genomes: Ouellet et al. [21] showed that functional miRNAs are processed from the HIV TAR element; Yeung et al. [22] have identified small non-coding RNAs in HIV infected cells and corroborated the existence of virally encoded miRNAs in nef and TAR; and Schopman et al. [23] have identified HIV-encoded small RNAs in virus-infected cells. While studies have reported that human endogenous retroviruses, retroelements and several exogenous retroviral sequences may have homologies with cellular miRNAs [24], the viral homologues of *human miRNAs in HIV-1 genomes* have not been identified and the role of miRNAs in HIV-1 infection has not been elucidated.

Our earlier proteomics and bioinformatics studies had identified a significant number of functionally relevant proteins that were upregulated, downregulated or produced *de novo* in a chronically HIV-infected CD4+ clonal T-cell line (RH9) [25], [26]. Based on these findings we hypothesized that the differential expression of proteins in HIV-infected versus uninfected counterpart cells is due to the dysregulation of cellular (or viral) microRNAs. Using multiple bioinformatics and computational tools we have identified 8 miRNAs that are predicted to bind to the mRNA targets of several proteins that are differentially expressed in HIV-infected T-cells (Table 1). To understand the biological significance of these miRNAs we searched for nucleotide sequence similarities between each of the miRNAs identified, and the whole or partial HIV-1 genome sequences present in the global databases. Herein, we report the discovery of *a sequence homologue* of hsa-miR-195 *within the HIV-1 protein-coding regions* of several HIV-1 strains from Africa (Table 2). In addition, we have identified sequence similarities between 3 other mature human miRNAs (hsa-miR-30d, hsa-miR-374a and hsa-miR-424) (Table 3) and one full-length miRNA (hsa-miR-30e) within the HIV-1 envelope regions (Table 4). A detailed search of the whole HIV-1 genome sequence also displayed similarity between 2 full-length human miRNAs (hsa-miR-30d and hsa-miR-424) and the gag-pol protein-encoding regions or pol only of 4 HIV-1 isolates (Table 4). These data have provided new insights into possible roles of viral env-gene associated miRNA-like sequences in the survival of HIV-1 genomes in the host, immune responses, virus replication, and pathogenesis.

**Table 1:** MicroRNA Sequences Used For Analysis.

microRNA	Length of miRNA : Full Length/Mature	miRBase Accession # : FullLength/Mature	Chromo-somal Location	Mature Cellular miRNA Sequence
hsa-miR-195	87 nt	MI0000489	17p13.1	**UAGCAGCACAGAAAUAUUGGC**
	21 nt	MIMAT0000461		
hsa-miR-15b	98 nt	MI0000438	3q25.33	**UAGCAGCACAUCAUGGUUUACA**
	22 nt	MIMAT0000417		
hsa-miR-16-1	89 nt	MI0000070	13q14.2	**UAGCAGCACGUAAAUAUUGGCG**
	22 nt	MIMAT0000069		
hsa-miR-16-2	81 nt	MI0000115	3q25.33	**UAGCAGCACGUAAAUAUUGGCG**
	22 nt	MIMAT0000069		
hsa-miR-30d	70 nt	MI0000255	8q24.22	**UGUAAACAUCCCCGACUGGAAG**
	22 nt	MIMAT0000245		
hsa-miR-30e	92 nt	MI0000749	1p34.2	**UGUAAACAUCCUUGACUGGAAG**
	22 nt	MIMAT0000692		
hsa-miR-374a	72 nt	MI0000782	Xq13.2	**UUAUAAUACAACCUGAUAAGUG**
	22 nt	MIMAT0000727		
hsa-miR-424	98 nt	MI0001446	Xq26.3	**CAGCAGCAAUUCAUGUUUUGAA**
	21 nt	MIMAT0001341		

**Table 2:** Sequence Similarity Between Human miR-195 and HIV Envelope Genes.

Gene Name/Description	Accession #/Subtype/Country of Origin		Alignment - Top line is hsa-miR195 (MI0000489) mature sequence, and bottom line is the env region of the respective virus.	Number and Percentage of Nucleotide Matches	SeedMatches
HIV-1 Isolate	GU216763	**1 tagcagcacagaaatatt 18**		18/18	6/6
#169b1a12	Subtype C	**||||||||||||||||||**		(100%)	100%
Envelope gene	South Africa	**1401 tagcagcacagaaatatt 1418**			
HIV-1 Isolate	GU216768	**1 tagcagcacagaaatatt 18**		17/18	5/6
#169b1c4	Subtype C	**|||| |||||||||||||**		(94%)	83%
Envelope gene	South Africa	**1404 tagcggcacagaaatatt 1421**			
HIV-1 Isolate	GU216773	**1 tagcagcacagaaatatt 18**		17/18	5/6
#169b1d7	Subtype C	**|||| |||||||||||||**		(94%)	83%
Envelope gene	South Africa	**1392 tagcggcacagaaatatt 1409**			
HIV-1 Isolate	HM215313	**1 tagcagcacagaaatatt 18**		17/18	5/6
#401-F1_8_10	Subtype CD	**||||| ||||||||||||**		94%	83%
Envelope gene	Tanzania	**1362 tagcaccacagaaatatt 137**			
HIV-1 Isolate	DQ199139	**1 tagcagcacagaaatatt 18**		17/18	6/6
#TZB0573	Subtype C	**|||||||||||||| |||**		94%	100%
Envelope gene	Tanzania	**585 tagcagcacagaaacatt 602**			

HIV-1 isolate #169b1a12, #169b1c4, and #169b1d7 are from the same patient.

**Table 3:** Sequence Similarity Between Three Mature Human microRNAs and HIV Envelope Genes.

Cellular miRNA (Mature Sequence)	Accession #/Subtype/Country of Origin	Gene Name/Description	Alignment - Top line is hsa-miR sequence, and bottom line is the env region of the respective virus.	Number and Percentage of Nucleotide Matches	Seed Matches
hsa-miR-30d	AY169802	HIV-1 Strain	**3 taaacatccccga 15**	13/13	5/6
	Group O	#98CMA104	**|||||||||||||**	(100%)	(83%)
	Cameroon	Complete	**6901 taaacatccccga 688**		
		genome			
hsa-miR-374a	AJ429907	HIV-1 Strain	**4 taatacaacctgataag 20**	13/17	4/6
	Group M	#00NE079	**|||||| | |||| ||**	(76%)	(67%)
	Subtype 6cpx	Envelope	**175 taataccaattgatcag 191**		
	Niger				
	AF391235	HIV-1 Clone	**2 tataatacaacctgataa 19**	17/18	6/6
	Group M	#TV006c9.1	**||||||||||| ||||||**	(94%)	(100%)
	Subtype C	Envelope	**542 tataatacaacttgataa 559**		
	South Africa				
hsa-miR-424	GU080167	HIV-1 Clone	**6 gcaattcatgtttt 19**	14/14	2/6
	Group M	704MC009F	**||||||||||||||**	(100%)	(33%)
	Subtype C	Envelope	**417 gcaattcatgtttt 404**		
	South Africa				

**Table 4:** Tabl**e 4.** Sequence Similarity Between Full Length Human microRNAs and HIV Genes.

Cellular miRNA	Accession#/Subtype/Countryof Origin	Gene Name/Description	Alignment	Number and Percentage of Nucleotide Matches	Seed Matches
hsa-miR-30e	FJ147129	HIV-1 Isolate	**1 tgtaaacatccttgactggaag 22**	17/22	4/6
	Subtype B	#VC2T2C1	**| |||| | |||||||||||**	77%	67%
	US	Envelope gene	**279 tttaaattgcgttgactggaag 300**		
	FJ147130	HIV-1 Isolate	**1 tgtaaacatccttgactggaag 22**	17/22	4/6
	Subtype B	#VC2T2C2	**| |||| | |||||||||||**	77%	67%
	US	Envelope gene	**273 tttaaattgcgttgactggaag 294**		
	U13543	HIV-1 Isolate	**1 tgtaaacatccttgactggaag 22**	18/22	5/6
	Subtype D	#93UG059	**| ||||| | |||||||||||**	82%	83%
	Uganda	Envelope gene	**45 tttaaactgcattgactggaag 66**		
	DQ208474	HIV-1 Isolate	**1 tgtaaacatccttgactggaag 22**	18/22	5/6
	Subtype AD	ML35.W0M.G2	**| ||||| | |||||||||||**	82%	83%
	Kenya	Envelope gene	**381 tttaaactgcattgactggaag 402**		
	DQ208473	HIV-1 Isolate	**1 tgtaaacatccttgactggaag 22**	18/22	5/6
	Subtype AD	ML35.W0M.F3	**| ||||| | |||||||||||**	82%	83%
	Kenya	Envelope gene	**381 tttaaactgcattgactggaag 402**		
hsa-miR-424	AM181808	HIV-1 Isolate	**33 gtgttctaaatggttcaaaacgtgaggcgctgctatac 70**	29/38	4/6*
	Subtype 13cpx	#01CMVP/CE	**|||||||||||||||| ||| | | ||||||||**	76%	67%
	Cameroon	Gag-Pol	**1049 gtgttctaaatggttctaaaattttcgtcatgctatac 1012**		
	GU207082	HIV-1 isolate	**33 gtgttctaaatggttcaaaacgtgaggcgctgctatac 70**	29/38	4/6*
	Subtype 13cpx	#VP_CE_104	**|||||||||||||||| ||| | | ||||||||**	76%	67%
	Cameroon	Pol gene	**817 gtgttctaaatggttctaaaattttcgtcatgctatac 780**		
	GQ344965	HIV-1 isolate	**33 gtgttctaaatggttcaaaacgtgaggcgctgcta 67**	27/35	4/6*
	Subtype AG	#06CM06BDH	**|||||||||||||||| ||| | || |||||**	77%	67%
	Cameroon	Pol gene	**520 gtgttctaaatggttctaaaattttggtcatgcta 486**		
hsa-miR-30d	GQ288251	HIV-1 isolate	**23 ggaagctgtaagacacag 40**	18/18	0/6
	Subtype B	#3077_051503	**||||||||||||||||||**	100%	0%
	US	Pol gene	**120 ggaagctgtaagacacag 103**		

* Represents seed matches which are in the reverse complementary strand.

## Results

### Identification of Cellular microRNA Sequences Associated with Dysregulated Proteins in HIV-Infected Cells

We used the program “GeneSet2miRNA” to identify potential microRNAs that could impact the activities of the proteins whose expression had been shown to be dysregulated in HIV-infected RH9 T-cells [25], [26]. For example, the microtubule-actin cross-linking factor 1 (MACF1) [27] is one of the proteins which we found was downregulated due to HIV-infection. Bioinformatics analyses indicated that the 3′ untranslated region of MACF1 has a sequence (5′ GGACAAUAGCUGCUA 3′) which is complementary to the seed sequence for the mature hsa-miR-195 (5′ UAGCAGCACAGAAAU 3′), indicating a strong binding affinity to this protein. MACF1 has an actin-regulated ATPase activity necessary for cross-linking actin to other cytoskeletal proteins. In addition, MACF isoforms act as positive regulators of Wnt receptor signaling pathway and are essential for controlling focal adhesions assembly. Actin-related proteins also play critical roles during HIV infection and latency in T-cells ([28] and our unpublished data).

Using an adjusted p-value of the enrichment (adjusted for multiple testing by Monte-Carlo simulations) cutoff of 0.05, we identified 7 microRNAs that could significantly bind to multiple mRNA targets in these cells. In addition, we selected one more miRNA from our initial search which fulfilled the criteria of being the best single-model match of the GeneSet2miRNA program. These 8 miRNA sequences are listed in Table 1. These analyses also revealed that hsa-miR-195 was one of the most frequently detected cellular miRNAs among the 8 miRNAs identified to be associated with mRNAs of the proteins modulated in our experimentally HIV-1 infected T-cells. Each of the miRNA and mRNA interactions were highly significant among 7 of the 8 miRNAs identified (p-value = 0.002 to 0.008).

### Discovery of miRNA-195 Homologues in the HIV-ENV Gene

Using both the mature (∼22 nucleotides) and full length (∼70 nucleotides) sequences of each of the 8 cellular miRNAs listed in Table 1 as query sequences, we have used multiple bioinformatics tools including BLAST (Basic Local Alignment Search Tool) and Clustal to align and scrutinize potential matches in HIV-1 genomes. An extensive search of more than 3000 complete HIV-1genomes and over 400,000 HIV-1 subgenomic sequences present in both the Los Alamos and NCBI databases resulted in thousands of potential matches. Each one of these matches was subsequently screened on the basis of the degree of homology and degree of sequence continuity with the full length and mature sequences of each of the 8 previously identified cellular miRNAs. These analyses resulted in the discovery of a perfect match (100%) from nucleotide positions 1 through 18 of the mature hsa-miR-195 sequence, including the seed region at nucleotide positions 2–7, within the envelope (*env*) protein-coding sequences of an HIV-1 isolate from South Africa (accession #GU216763) (Table 2). Two other isolates, #GU216768 and #GU216773, from the same individual also showed homology with hsa-miRNA-195 in the same region of the Env gene and again matched miR-195 at positions 1–18, with 17 of 18 (94%) nucleotides matching perfectly (Table 2). This single nucleotide mutation (i.e. 17 versus 18 nucleotides) represents the genetic diversity due to mutations known to be present in *all* HIV-1 envelope genes of both acute and chronically HIV-1 infected individuals [29]. In addition to the sequence homology in the South African HIV-1 isolates (#GU216763), we have identified two other strains from Tanzania, #HM215313 and #DQ199139, whose Env gene sequences also matched with the same region of hsa-miR-195 at 17 of 18 nucleotide positions, as in the GU216763 isolate (Table 2).

### Identification of HIV-ENV Gene Homology Domains in Other Cellular microRNAs

Analyses of the remaining seven cellular miRNAs that were predicted to be associated with multiple dysregulated proteins expressed in our experimentally HIV-infected cells (Table 1) also showed sequence similarities between 4 microRNA sequences and the *env* regions of several other HIV-1 strains (Tables 3, 4). Each of the seven miRNAs were screened using the BLAST algorithm to find the best matches within all the complete and partial HIV-1 genome sequences contained in both NCBI and the Los Alamos HIV databases. Again, several thousand preliminary matches were screened for the degree of homology, degree of continuity and presence of the seed sequence. These analyses indicated that in addition to hsa-miR-195, there are three other cellular mature miRNAs (hsa-miR-424, hsa-miR-374a, hsa-miR-30d) that displayed 13–18 nucleotide length homologous regions within the Env genes of four different HIV-1 strains (Table 3). We have also tested full-length (∼70 nucleotides) miRNAs and found that hsa-miR-30e exhibited sequence homology within the *env* regions of 5 different HIV-1 isolates (Table 4). These sequences were distinct from those aligned with miR-195 and were located in different regions of the HIV-1 envelope than hsa-miR-195. Thus, the number, length, and percentage of matches within the seed region of each miRNA and their respective HIV env-gene sequence were variable.

### Identification of miRNA-Homology Domains in other Regions of HIV-genomes

To determine if any other region of the HIV-1 genome might show homology domains, all the preliminary alignments were screened again using the query sequences of each of the 8 cellular miRNAs that we had identified earlier. Our results indicated that 2 out of the 8 predicted miRNAs (hsa-miR-424 and hsa-miR-30d), showed homologies in different regions within the HIV-1 genome (Table 4). Both full-length cellular miRNAs (hsa-miR-424 and hsa-miR-30d) exhibited 76%–100% homology domains within the gag-pol regions of four isolates from three HIV-1 strains (Table 4). These findings suggest that multiple cellular miRNAs have homology domains in different regions of various HIV-1 genomes (Tables 2, 3 & 4). Thus, out of the 8 miRNAs that we identified for further analysis, 5 (hsa-miR-195, hsa-miR-424, hsa-miR-30d, hsa-miR-30e and hsa-miR-374a) showed homology domains in the HIV-1 genomes of several strains, and 3 (hsa-mir-15b, hsa-miR-16-1 and hsa-miR-16-2) did not.

### Clustal Analyses and Mapping of MiRNA-like Sequences

Since the most significant matches in our study occurred between the cellular miRNA hsa-miR-195 and the *env* protein encoding regions of HIV-1 isolates from Africa, we further scrutinized and characterized these sequences by aligning the genomes of different HIV-1 clades from various regions around the world with the hsa-miR-195-like sequence homology domains. Using the ClustalW2 algorithm, as well as the Los Alamos compendium of all HIV-1 alignments, we were able to show the exact regions of these sequences in each of the 6 clades that corresponded to the position of the hsa-miR-195-like sequence we have identified from the African HIV-1 strains (Table 5).

**Table 5:** Sequence Homology Domains in the V5 Regions of HIV Envelope.

Description	hsa-miR-195 Sequence	Matches	Accession
	Alignment With V5 Regions		
hsa-miR-195	TAGCAGCACAGAAATATT		
	||||||||||||||||||		
M-Clade C	TAGCAGCACAGAAATATT	18/18	GU216763
M-Clade C	TAGCGGCACAGAAATATT	17/18	GU216773
M-Clade C	TAGCGGCACAGAAATATT	17/18	GU216768
M-Clade C	TAGCACCACAGAAATATT	17/18	DHM215313
M-Clade C	TAGCAGCACAGAAACATT	17/18	DQ199139
M-Clade C	TAGCACAAAAGAGATATT	14/18	U46016
M-Clade G	TAGCACTAGTGAGATCTT	12/18	AF084936
M-Clade B	GAACCAGACCGAGATCTT	11/18	U21135
M-Clade B	TAATGACACCGAGGTCTT	10/18	K02007
M-Clade E	TGAGACCATCGAAACCTT	10/18	AF197341
M-Clade B	TAATGATACCGAGACCTT	9/18	AF004394
M-Clade B	–AAGACACTGAGATCTT	9/18	AF042101
M-Clade A	TACAAAAAATGAGACCTT	9/18	M62320
M-Clade A	CAGTGAACCTGAAACCTT	9/18	AF484509
M-Clade A	CAATGTAAATGAAACCTT	8/18	AF004885
M-Clade E	TGCGACTAATGAGACCTT	8/18	AF197340
M-Clade D	–GTACTAACGAGACCTT	8/18	K03454
M-Clade B	–ATGGGTCCGAGATCTT	8/18	K02013
M-Clade B	–ATGAGACCGAGACCTT	7/18	D10112
M-Clade B	–ATGAGTCCGAGATCTT	7/18	AF033819
M-Clade B#	–ATGAGTCCGAGATCTT	7/18	K03455
M-Clade B	–ATGAGTCCGAGATCTT	7/18	D86069
	** **		

Selected alignments of V5 regions of 22 different.

HIV-1 strains containing the hsa-miR-195-like sequence. The hsa-miR-195 sequence.

was first aligned to the 5 African strains (shaded and also shown in Table 2), and.

then aligned with 17 representative HIV strains from different.

clades as determined by the ClustalW2 algorithm. The mismatches between the clades are due to.

the variability of the HIV envelope sequences, including V5 regions.

# Represents the HIV HXB2 strain which was used for gene mapping in Figure 2.

Asterisks represent positions of 100% conservation.

We chose 17 HIV-1 genomes that had been identified as *representative strains* from each of the 6 HIV-1 clades (A, B, C, D, E and G) to perform alignments, and used whole genome sequences to produce the best alignment. These analyses revealed that the hsa-miR-195-like region that we have identified aligns best to a specific region in the Env gene of African strains (Table 5, shaded area). However, while miR-195-like sequences were shared by all representative strains among the 6 clades that we examined, these sequences were highly divergent among the different clades (Table 5).

**Figure 1:**
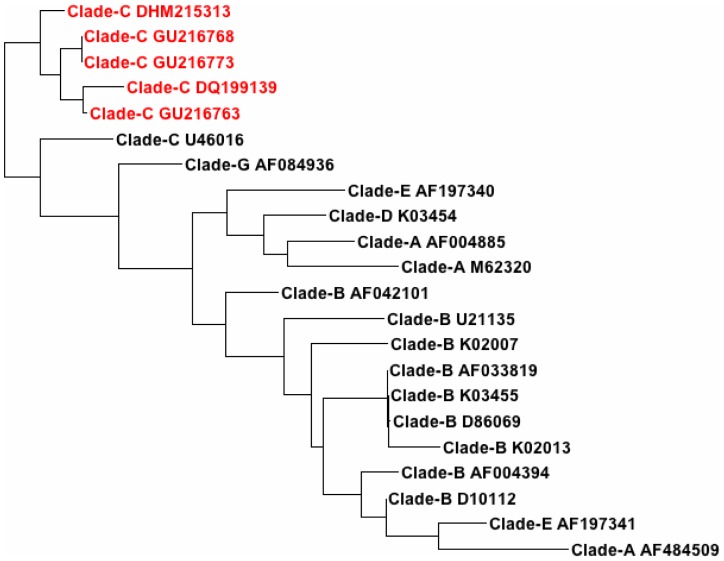
Phylogenetic Tree of miR-195-like sequences in different HIV clades. This figure shows the envelope region of the HIV HXB2 genome to which the hsa-miR-195-like seqeunce maps. The area corresponding to the V5 region of HXB2 spans nucleotide positions 7603 through 7632 and is highlighted in yellow. The hsa-miR-195-like region is shown in blue. The mismatches between the HXB2 sequence and the miR-like sequence present in the African strain #GU216763 are due to the sequence variations in envelope regions of all HIV genomes.

In addition to defining the specificity of sequence alignments, we used the TreeDyn software for the construction of a sequence-based relational tree using the alignment data generated by the Clustal algorithm. As can be seen in Figure 1, all viral genomes that shared miR-195-like sequences are clustered together in Clade C.

**Figure 2:**
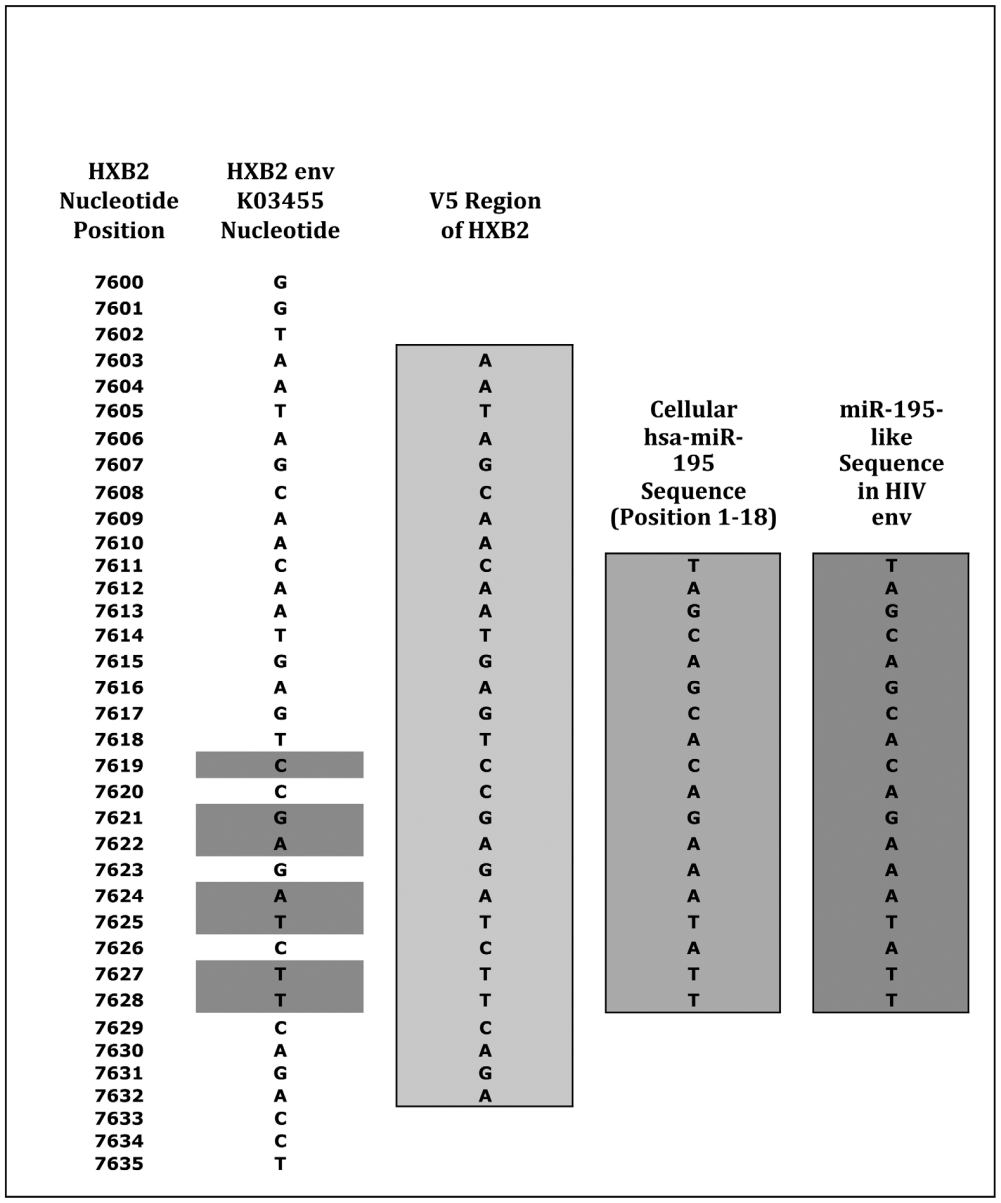
Location of hsa-miR-195-like Sequence in HXB2 Env Gene. Phylogenetic tree was constructed using the hsa-miR-195 sequence TAGCAGCACAGAAATATT for alignment similar to that used in Table 5. This tree was constructed using the treedyn program. Each leaf represents one of the 5 African microRNA-like sequences or one of the 17 reference strains from the 6 clades which were used for alignment. The five sequences highlighted in red represent the 5 African microRNA-like sequences identified in Table 2.

While detailed maps of the *env* region exist for several HIV-1 strains, there is no map at present for the HIV-1 genomes from Africa in which we found the greatest homology. We therefore cross-referenced our sequence with the HXB2 strain (Clade B) for which a detailed map is available (GenBank accession number K03455). The HXB2 genome is used as a common reference strain for many different functional studies, and it has a standardized position numbering scheme available at the Los Alamos National Laboratory website (http://www.hiv.lanl.gov/). The alignment given in Table 5 can be cross-referenced with the nucleotide-specific map of the HXB2 strain and indicates that the ‘hsa-miRNA-195-like’ sequence maps to the segment from position 7611 through 7628 within the *env* gene of the HXB2 genome (Figure 2). It is interesting to note that Yeung et al [22] show a small RNA species in their supplementary table at position #7601 which corresponds to our 195-like sequence, albeit with less homology (because they used clade B),(Table 5). This region corresponds to the V5 region of the envelope glycoprotein. As can be seen from Figure 2, the miRNA-195-like sequence is embedded *entirely within the V5 region of the HIV-1* genome.

**Figure 3:**
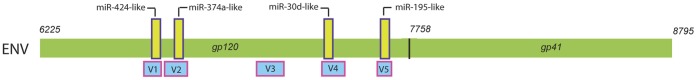
Localization of miR-like sequences in HIV envelope variable regions. This figure shows the miR–like domains listed in Tables 2 and 3 and mapped to their corresponding positions in the HXB2 envelope gene (at nucleotide positions 6682–6694, 6765–6782, 7386–7398 and 7611–7628). The miR-424-like, miR-374a-like, miR-30d-like, and miR-195-like sequences are embedded in the hypervariable regions corresponding to V1, V2, V4 and V5 respectively and are depicted in blue. No miRNA-like sequence was detected in the V3 region of the envelope.

Next, we used the Clustal program to similarly align the homologous regions of hsa-miR-424, miR-374a, and miR-30d (Table 3) to the HXB2 genome. Our results show that the ‘hsa-miR-424-like’ sequence maps inside the V1 region of the HXB2 env gene at nucleotide positions 6682–6694, ‘hsa-miR-374a-like’ maps inside the V2 region (position 6763–-6781), and ‘miR-30d-like’ lies inside the V4 domain at positions 7386–7398 (Figure 3). There was no homologous domain present in the V3 region to any of the miRNAs that we examined.

### The miRNA-like Sequences are not a Classical Human Cellular miRNA

To determine if the ‘miR-like’ sequences that we identified in the HIV-1 envelope gene are of viral or cellular origin, we searched the entire NCBI human genome sequence database for possible homology domains anywhere. Using the BLAST algorithm and the 18-nucleotide miR-195-like sequence as the query, only one significant match was localized to nucleotides 6524363 through 6524380 (18/18 matches) on the human chromosome 17 (Table 6). While this region corresponds to the published location of the 21 nt hsa-miR-195 sequence [30], our HIV-env-associated miR-195-like sequence is not that of a typical miRNA because: 1) this sequence encodes a functional protein; 2) it is not a part of a stem-loop structure necessary for the processing of miRNAs; 3) there is no evidence that the miR-195-like sequence is processed from a long precursor transcript (pre-miRNA) by Drosha; and 4) there is no evidence that this sequence is cleaved into mature miRNA by Dicer enzyme. Further, it appears unlikely that the newly discovered miR-195-like sequence in the HIV genomes could be incorporated into RISC complexes that guide the miRNA to the mRNA target site.

**Table 6:** MicroRNA-like sequence alignments with cellular genome sequences.

miR-195-like:	1 TAGCAGCACAGAAATATT 18
	||||||||||||||||||
Human chr 17:	6524380 TAGCAGCACAGAAATATT 6524363
miR-30d-like:	1 TAAACATCCCCGA 13
	|||||||||||||
Human chr 8:	49090730 TAAACATCCCCGA 49090718
miR-374a-like:	1 TATAATACAACCTGATAA 18
	||||||||||||||||||
Human chr X:	11825168 TATAATACAACCTGATAA 11825151
miR-424-like:	1 GCAATTCATGTTTT 14
	||||||||||||||
Human chr X:	17948436 GCAATTCATGTTTT 17948423

BLAST search result and alignment using miR-195-like sequence against the human genome sequence and locations on the chromosomal DNAs.

Regardless of these controversies, Yeung et al [22] first suggested that small RNA molecules in HIV-infected cells may represent products of Dicer cleavage. Subsequently, a high-throughput deep sequencing study of siRNA from HIV-infected cells suggested that the viral dsRNA intermediates may be processed by Drosha and Dicer [23]. However, the small RNAs found in these studies are non-coding and preliminary transfection of these “viral microRNA” clones did not show significant changes in virus production [23]. In addition, the miRNA-like sequences we have discovered are not related to those reported in any published studies.

The other miR-like sequences were also similarly localized to their genomic regions using the BLAST algorithm (Table 6). The results show that the miR-like sequences are most significantly matched to the position in the human genome that corresponds to the actual cellular miRNA regions. However, the BLAST searches showed several less significant matches of miR-like sequences in other parts of the human genome, but these were not 100% matches.

## Discussion

The potential involvement of miRNAs in HIV-1 proliferation and life cycle is the subject of much research. While the dysregulation of miRNA expression in HIV-1 infected cells has been known since 2005 [12], studies have now identified HIV-TAR and Nef-LTR regulating miRNAs [21], [22]. In addition *target sites* have been predicted for cellular miRNAs such as *nef-LTR* regions in HIV-1 genomes [10], indicating that human cellular miRNAs can modulate HIV-1 expression and replication [13]. Yeung et al [22] had first suggested that small RNA molecules in HIV-infected cells may represent products of Dicer cleavage. Subsequently a high-throughput deep sequencing study of siRNA from HIV-infected cells suggested that the viral dsRNA intermediates may be processed by Drosha and Dicer [23]. However, the small RNAs found in these studies are non-coding and preliminary transfection of these “viral microRNA” clones did not show significant changes in virus production although some small RNAs showed some inhibition [23]. Thus, the miRNAlike sequences we have discovered in HIV genomes are not related to those reported in any published studies.

HIV-1 genomes can interact with miRNA in two ways: direct binding of a cellular miRNA with a viral transcript or the RNA genome itself, or indirectly, by interacting via a host-cellular factor that is required for HIV-1 infection and viral life cycle. While most miRNAs regulate gene expression by suppressing their target mRNAs, a cellular miRNA could target host factors, which either suppress or enhance HIV-1 infection. Also, a single cellular miRNA can target as many as 100 transcripts. This makes it likely that any given cellular miRNA involved in HIV-1 infection is probably serving as both an activator and a suppressor of HIV-1 at the same time. While the multitude of interactions inside a cell are extremely complex and dynamic for determining the exact role of a given cellular miRNA as purely an up- or down-regulator of HIV-1 infection, there is evidence for both direct and indirect regulation of cellular and viral gene expression by cellular miRNAs [31].

We have conducted a thorough literature search for the possible biological functions of cellular hsa-miR-195, miR-30d, miR-424 and miR-374a as it relates to HIV infection. A total of 17 papers were found to report on a wide range of functionalities of miR-195, 6 citations were associated with miR-30d function, 8 papers discussed miR-424 gene function and only 1 citation described miR-374a function. Most papers associate these microRNAs to cancer, apoptosis, Alzheimer’s disease and signal transduction [32]–[35] and none was related to any of the miRNAs expressed during HIV infection.

The miRNA-like sequences we have identified in HIV-1 are unique in that they do *not* seem to be derived from cellular miRNA, nor do they appear to represent viral miRNAs or their targets. The hsa-miR-195-like sequence corresponds to the first 18 nucleotides of the mature hsa-miR-195, which has a length of 21 nucleotides. Any functional similarity of this sequence to the cellular hsa-miR-195 may be speculative. However, it should be noted that there are miRNAs as short as 17 nt; and that HIV-1 has been reported to encode a viral miRNA, designated TAR-3p, whose cloned length is 17 nt [20]. Further, the potential action of a miRNA is mostly dependent on base pairing between the miRNA seed sequence and its target; positions 13–16 of the miRNA may aid in pairing as well [5], [36]. The miR-195-like sequence we have identified in #GU216763 contains both the seed region and positions 13–16 of hsa-miR-195 and is 100% conserved in these regions. It has also been predicted computationally that the cellular hsa-miR-195 may interact with the HIV-1 Nef in the3’ LTR region based on a perfect complementarity of a *7 nucleotide seed sequence* with its viral target [31].

Whether the microRNA-like sequences are of viral origin or products of provirus integration millions of years ago remains to be explored. However, the fact that this sequence is part of a functional coding region of a vital viral gene makes the integration event scenario seem unlikely. Our data suggest that the miR-like sequences present in the HIV-1 envelope region are *viral RNA sequences, which emulate a cellular miRNA*.

The implications of a viral miRNA mimicking a cellular miRNA would be speculative. However, there is precedence for a viral miRNA to outcompete its cellular competitor. HIV-1 TAR RNA has been reported to act as a sort of miRNA-‘decoy’, decreasing the host cell’s RNAi activity by binding and sequestering TRBP, a TAR RNA-binding protein and an essential Dicer-cofactor [37].

We therefore propose that the hsa-miR-like sequences we have identified in the Env genes of several viruses may similarly be titrating out the related cellular miRNA targets.

A major point of distinction between miRNAs and our newly identified miRNA-like sequences is that while cellular miRNAs are derived from *non-coding* regions of the DNA, the miRNA-like sequences we have identified are located in the *coding* regions of vital HIV-1 genes. The HIV-1 envelope glycoprotein contains 5 variable regions (V1–V5) interspersed by conserved regions C1–C5. The miRNA-like sequences we discovered have been mapped to the V1, V2, V4 and V5 regions of the HIV-1 envelope and are integral components of the HIV-1 gene, which codes for a functional envelope gp120 (Figure 3). The finding of several viral sequences homologous to cellular miR-30d, miR-30e, miR-374a, miR-424 and miRNA-195 in different regions of the HIV-1 genome but primarily in the envelope regions of several HIV-1 strains indicate that the phenomenon of cellular miRNA-like sequences in the HIV-1 genome may be widespread.

Changes in the length of amino acid sequences or glycosylation patterns in the variable V-regions are critical to HIV-1 infection because they can affect not only the cellular tropism but can also modulate sensitivity to virus neutralization and disease progression [38]. Some of the major determinants that contribute to biological activities of HIV-1 strains including replication, viral tropism (ability to infect T-cells versus macrophages or other cell types), sensitivity to neutralization, modulation of the CD4 antigen, and cytopathogenicity are localized in the V1 to V5 regions of the HIV-1 envelope glycoprotein gp120 [39]–[41]. These sequences are critical for effective humoral responses and virus neutralization. The V1 toV5 regions of the HIV-1 envelope have been associated with the rate of replication, virus neutralization and pathogenesis of HIV-1 strains [42], [43]. While the primary virus replicates in the body, it becomes resistant to neutralization because some of the specific V1 to V5 domains are either lost or have been modified by increased numbers of N-linked glycosylation sites and therefore cellular immune responses to HIV-1 infection are compromised [39]–[41].

Finally, our findings of four different miRNA-like sequences within the V1, V2, V4 and V5 regions of the HIV-1 *env* gene may provide new insights that will contribute to a better understanding of the molecular complexities of HIV-1 infection and pathogenesis. The miRNA-like sequences may emulate a non-coding cellular miRNA and therefore could represent the first examples of *human cellular homologues of* miRNAs in HIV-1 coding regions. These sequences may therefore play a role in HIV-1 replication, immunity, and virus neutralization, and thus may influence pathogenesis in HIV-infected individuals. Detailed *in vivo* studies and construction of vectors containing the human microRNA-like sequences that we have discovered, would yield critical results that would allow us to develop a humanized mouse model system to test the effects of these vectors *in vivo*. These experiments will demonstrate the influence of microRNA-like sequences on virus replication, as well as antibody and antigen production *in vitro* and *in vivo*.

## Materials and Methods

### Source of Data

Previous proteomics and bioinformatics research in our laboratory had identified >200 differentially expressed, functionally relevant proteins in an HIV-1 infected CD4+ T-cell line (RH9) analyzed sequentially over a period of approximately 2 years [25], [26]. In this study, we used GeneSet2miRNA [44] to identify potential microRNAs that could impact the activities of our differentially regulated proteins. Using an adjusted p-value of the enrichment (adjusted for multiple testing by Monte-Carlo simulations) cutoff of 0.05, we identified 7 miRNAs that may significantly bind to multiple mRNA targets. We also selected one other miRNA because it was identified as the best single-model match. These 8 miRNAs are listed in Table 1.

### Identification of Homologous Sequences

To identify sequences that could be homologous to HIV-1, we downloaded the *full length and mature sequences* of the 8 human microRNAs from the miRBase database (http://mirbase.org/) that had been shown to be significantly associated with the proteins modulated by HIV-infection of CD4+ T-cells. Each of the 8 miRNAs was used as a separate query, utilizing both the mature and full-length versions of each miRNA. The BLAST (Basic Local Alignment Search Tool) [45] program was used to search against the entire HIV-1 databases (http://blast.ncbi.nlm.nih.gov/) (HIV taxid: 11676) at the National Center for Biotechnology Information (NCBI) and the Los Alamos HIV databases (http://www.hiv.lanl.gov/content/sequence/BASIC_BLAST/basic_blast.html). All full-length and partial HIV-1 genome sequences, representative of all HIV-1 clades and strains, were used for the analyses. These sequences have been identified by the International Committee on Taxonomy of viruses (ICTV) and are available in the global public databases. The outputs from both database searches were compared and the best matches from all microRNA query searches were selected based on the length of the match, percentage of identity of match, lack of gaps or deletions, and inclusion of the seed sequence.

### Clustal Analyses and Mapping of Newly Identified Sequences

The Clustal algorithm was used for multiple sequence alignments [46], [47] (http://www.ebi.ac.uk/Tools/msa/clustalw2/). We used the five most homologous HIV-1 sequences to hsa-miR-195 as identified by our BLAST searches and shown in Table 2, as well as the sequences of 17 other representative HIV-1 strains from 6 clades to generate alignments using the Clustal algorithm. Complete genome sequences from each of the representative HIV-1 strains were used to perform alignments of the different clades with our best matches. Results from the Clustal algorithm were then checked against the Los Alamos HIV Compendium (http://www.hiv.lanl.gov/content/sequence/HIV/COMPENDIUM/compendium.html) to verify that the alignments from both sources were in agreement.

In addition to defining the specificity of sequence alignments, we used the TreeDyn software program for the construction of a sequence-based relational tree using the alignment data generated by the Clustal algorithm (http://www.treedyn.org/). The target regions of the alignment were then mapped to the HXB2 strain gene map using the Los Alamos National Laboratory HIV genome database (http://www.hiv.lanl.gov/) map, because this is one of the most complete reference sequence data maps available for HIV-1.
